# Chicago Gynecological Society

**Published:** 1884-12

**Authors:** W. W. Jaggard

**Affiliations:** 2330 Indiana ave.


### SOCIETY PROCEEDINGS.

---

CHICAGO GYNECOLOGICAL SOCIETY.

*October 31, 1884. Regular Meeting.*—In conformity with an ancient custom, the members of the Society met at the residence (271 Michigan avenue) of the retiring President, Dr. A. Reeves Jackson, and were entertained by an elegant and elaborate banquet. After the banquet, the President called the meeting to order. The following officers for the ensuing year were elected:

President, Dr. H. P. Merriman; 1st Vice-President, Dr. E. C. Dudley; 2nd Vice-President, Dr. Charles Warrington Earle; Secretary and Treasurer, Dr. Edward Warren Sawyer; Editor, Dr. W. W. Jaggard.

After an appropriate address by the retiring President, the Society adjourned to meet Friday, Nov. 21, at the residence of Dr. C. W. Earle, 535 Washington boulevard, Dr. Edward Warren Sawyer to open the discussion of "The Premature Expulsion of the Ovum."

*Address of the Retiring President*—A. REEVES JACKSON, M.D.—In the course of his annual address, the retiring President, Dr. A. Reeves Jackson, in alluding to various influences which had retarded the progress and lessened the usefulness of the society, said: "This tendency to decay is especially manifest to those of us who remember the enthusiasm which characterized its earlier meetings. Then, the essayist was always present to fill his appointment. The other members, equally diligent, came promptly, each filled to the brim with the subject for the evening's study, and ready to bubble over, like a boiling tea-kettle; and knowledge fairly oozed out of them, as Mark Twain once said of himself, "like the attar of roses out of the—otter." Those were halcyon days! The meetings were looked forward to with as much pleasurable anticipation as is felt by the boy who gazes upon the marvelous posters of a coming circus. This zeal so pervasive, and so promising of good results, came, by and by, to lessen; the early, lover-like ardor, began clearly to show signs of cooling. The transactions of the society possessed less and less of interest; the attendance was less regular, and less constant. The brightness of our sun has dimmed; there remains only the afterglow. There came a time when we all realized that the health of the society was failing. The pathology was complicated; but these conditions at least were present—anemia, atrophy, nervous exhaustion. Occasionally, a little fresh, healthy blood was infused, and under the stimulus the patient's drooping energies and failing strength were temporarily revived; but, mingling as it did with so large a proportion of the old, its influence was rarely felt beyond a single night, and the entire mass became as stagnant as before.

What shall the treatment be? While I desire council, I venture to suggest, for your consideration, a few remedies, calculated, as I believe, to meet some of the indications.

1. *For the Anemia.*—We need a largely increased membership. In order to attain this three things seem necessary:

1. To make the Society attractive.
2. To make its attractions known.
3. To make admission easy.

In regard to the first of these I have to say that the attractiveness of a scientific society can only depend upon the personal interest felt in, and given to the proceedings by those who take part in them. Therefore, every member should add his thoughts and his voice; and he should, by preparation, if necessary, add them intelligently.

But, next to having an attractive stock of goods, the wise merchant informs the public of the fact, and invites inspection. Let us imitate him.

Prior to each meeting let every one of us invite some one or more of our professional friends to be present, assuring them of a cordial welcome. We are thought to be a very exclusive society. Let us remove that impression. Not only should we invite reputable members of our profession to visit us, but we should invite them to join us. Very many persons who would never *seek* admission to a society would gladly enter if they had assurance that their membership was desired.

There is an inspiration in mere numbers—and we need it.

To make admission easy, abolish the requirement of an inaugural paper. This is a stumbling block to many; and to all, the trial is a severe one. The knowledge that the essay is to be a *test;* that it is to be *criticized,*—perhaps adversely,—is very likely, nay, in most cases, very certain to deter even those who would become valuable members from making application for membership. I am aware that we want workers; but we also want listeners. We want producers, but we also want consumers. If a man will not or cannot write a paper, he can listen to one. If he cannot instruct, he may be instructed. If he cannot do us good, we may still benefit him. And if we added a score of non-workers to our membership, the number of workers would not be thereby diminished. But it cannot be doubted that from an increase of membership obtained, in the way I have suggested, a certain proportion would become active and efficient.

2. *For the Atrophy.* This will be benefited by the increased membership. The body grows in proportion as it is supplied with blood. So does each of its parts; and as the characteristics of a community are the results of those of its individual constituents, each of these latter must be looked to. Each individual—each part—must grow, and then we secure growth of the whole.

3. *For the Neurasthenia.*—This condition is functional only, dependent doubtless upon the two which I have named. In this case, as in many others I have seen, it is not the result of over-work; not so much exhaustion as lack of energy; not so much debility as indisposition.

The rest treatment has been tried and has failed. Let us try the effect of exercise.

It only remains for me to thank the members of the Society for the undeserved honor conferred upon me one year ago, and I have finished.

In retiring from the presidency, I desire to express the hope that when, a year hence, my successor shall address us, and concerning the then future of our Society shall ask the question:

"Watchman! tell us of the night,
What its signs of promise are."

A respondent, pointing to our lengthened roll, our increased and improved work, may be able to say:

"Traveler! From yon mountain height,
See that glory-beaming star!"

---

*Friday, Nov. 21, 1884, Regular Meeting.*—The President, H. P. Merriman, in the chair.

Dr. Edward Warren Sawyer opened the discussion on the "Treatment ot Abortion." Dr. Sawyer called attention to the frequency of the interruption of pregnancy before fœtal viability. Madame La Chapelle says abortions are as frequent as labors at full term. The experience of the profession opposes this generalization. In his own practice, extending through a period of ten years, he had only seen from forty to forty-five abortions. As illustrative of the wonderful conservatism and care of nature in these cases, he had never met with a fatal case in his own practice, and had seen but one fatal case in the practice of his medical brothers. This fatal case was complicated by cellultis and pneumonia.

As to causation, abortions are divisible into two classes: (1) those occasioned by natural processes; (2) those induced by accidental or intentional violence. The former class usually terminates favorably, the latter class is the *bête noir* of the physician. It is a matter of medico-legal interest, that in abortions resulting from natural processes, *i. e.*, fatty degeneration of the decidua, the ovum and decidua are expelled as a rule, in an intact condition, while in criminal abortion, the product of conception is expelled in a more or less mutilated state. When the ovum has been mutilated, the embryo is extruded from the cavity of the uterus before the fœtal envelopes and decidua, and is frequently lost. Moreover, the embryo, so late as the fourth week, may be completely absorbed. The absence of the fœtus cannot be regarded in the differential diagnosis of abortion, molar pregnancy, and intra-uterine fibroids. Two distinct courses, as to the treatment of abortion have been adopted by the profession. The radical method of immediate evacuation of the contents of the uterine cavity, and the plan of patient waiting have been, in turn, defended and opposed. Dr. Sawyer has followed the expectant mode of treatment. He waits and allows nature to effect the expulsion of the product of conception. He has waited as long as one week. He has been encouraged in this line of treatment by the fact that he has never seen any untoward consequences in the cases managed in this manner. He enjoins absolute rest in the horizontal position, and the exhibition of quinine and alcohol, as required. Local treatment, apart from the vaginal tampon, is limited to vaginal injections of chlorinated soda. He has never noticed hemorrhage or inflammatory action as the result of this course of action. He objects to the removal of the whole or part of the product of conception from the uterine cavity, because, (1) it is a painful procedure, involving the use of ether, which predisposes to hemorrhage from uterine inertia; (2) an assistant is necessary; (3) the amount of unavoidable injury to the genital tract is considerable.

In conclusion, Dr. Sawyer exhibited an unusual specimen. The specimen consisted of an intact, amniotic sac, enclosing a five months' fœtus, with velamentous insertion of the umbilical cord. Separation had occurred between amnion and choroion. The chorion, placenta, and decidua, remained within the cavity of the uterus. He had been called to see the woman, five months advanced in pregnancy, who was suffering from severe uterine contractions and hemorrhage. After a brief interval, the specimen, presented to the society, was expelled. Noting the intact condition of the amnion, Dr. Sawyer paid no further attention to the mass, told the woman to fear no more trouble, and went home. Next morning, upon visiting his patient, he was informed, that one hour after his departure, under renewed hemorrhage and uterine contractions, another mass was expelled. This second mass proved to be the chorion, placenta, and decidua. A medical friend, Dr. Albert G. Paine, had observed a strictly similar case.

*Discussion.*—Dr. W. W. Jaggard said separation between the amnion and chorion was relatively infrequent during the sixth and seventh months, but was not uncommon prior to that period. In regard to the treatment of inevitable abortion, when the ovum was expelled in an intact or mutilated condition and decidua or portions of the fœtal membranes remained within the uterine cavity, it was necessary to regard the natural history of the condition. This condition had been appropriately termed by Breslau "incomplete abortion." The terminations are briefly:

(*a*) Spontaneous elimination of that portion of the product of conception remaining within the uterine cavity, as the result of retrograde metamorphosis, accompanied by intermittent hemorrhages and uterine contractions.

(*b*) Sometimes,—though seldom,—hemorrhage ceases entirely and the patient is apparently well. This interval varies from a few days or weeks to months. Suddenly hemorrhage and pains recur and the intra-uterine mass is expelled. This retention, with a long interval of rest, is noticed when the placental or decidual attachments are intact. That this act constitutes the termination of the labor, so to speak, is apparent from the fact that the milk secretion is usually established at this time, and the reductive metamorphosis of the uterus is instituted.

(*c*) More frequently, the retained decidua, or placenta, undergoes suppurative or ichorous changes, as the result of which systemic infection is liable to occur, despite the thrombosis of the uterine sinuses, and the proliferative change, in the uterine mucosa.

(*d*) The retained placenta or decidua becomes converted into placental or fibrinous polyps—conditions, which always require operative interference.

All four terminations present sources of danger to the mother. From this glance at the natural history of the condition,—for the elucidation of which Spiegelberg deserves especial recognition,—the weight of evidence is in favor of the so-called radical treatment. Empty the cavity of the uterus at the earliest possible period. The plan recommended by Dr. Mundé, in the February number of the *American Journal of Obstetrics*, 1883, was worthy of high commendation. One finger within the uterus, one hand on the fundus was preferable to instruments, when equally effective. The subsequent treatment was one of extreme importance.

Whenever the cavity of the uterus is invaded by the finger, or any instrument, it must be irrigated by some antiseptic solution. Two per cent. solutions of carbolic acid, or one to two thousand of the bichloride of mercury, are efficient in the prevention of conditions, favoring decomposition and sepsis. After irrigation of the cavity of the uterus, it was a good plan to introduce within the uterus a *bacillus* of powdered iodoform, weighing at least six grammes. Symptoms of iodoform intoxication rarely, if ever, followed the exhibition of this quantity. Ten grammes are usually required to produce toxæmia.

Dr. Philip Adolphus said that abortions were more frequent among multipara than primipara. In the way of prophylactic treatment, he thought women ought to sleep by themselves, during the time corresponding to their menstrual periods. Among the upper classes in Europe, it is customary for man and wife to sleep in separate beds. He thought it an excellent plan. When abortion was inevitable, the treatment must be symptomatic. To arrest hemorrhage, plug the cervix, not the vagina. Use tupelo, or laminaria tents, not those composed of sponge, for obvious reasons.

Use thin tents. When fetor is noticed, empty the uterine cavity. For this purpose, either finger or curette might be employed.

Dr. D. T. Nelson said the dangers from abortion were (1) hemorrhage; (2) sepsis; (3) inflammation. Rest and opium were not sufficient. He could not rest until the uterine cavity was empty. He had no sympathy with the expectant plan of treatment. The manner of emptying the uterine cavity was important. If the cervix was dilated, or dilatable, the cavity should at once be cleaned out with the finger. If the cervix was not dilated, nor dilatable, the cervix ought to be plugged in the manner indicated by Dr. Adolphus, with tupelo, or laminaria tents. If the cervix was partially dilated, or dilatable, and the uterus fixed, give an anæsthetic, relax the spasm, and proceed as in the first case. He had no fear of ether predisposing to uterine inertia. In dissecting off the placenta, it was advisable to glove the finger tips with the amnion. In the early months, when the ovum was attached near the cornua, it was necessary to bear in mind the possibility of irregular contraction, and the inclusion within either cornu of a bit of the placenta. The cornua must be thoroughly explored. When he employed intra-uterine irrigation,—by no means an invariable method of treatment,—he used a one-half per cent. sol. of carbolic acid, or a dilute. solution of ordinary table salt. The solution must be hot, 110°–120°F. Hot water, in the absence of carbolic acid, or salt, was effective as a cleansing agent, and as inducing uterine contractions. Dr. Nelson had had no experience whatsoever with iodoform, but regarded it as superfluous in all cases.

Dr. Wm. E. Clarke was more afraid of hemorrhage and sepsis, than of inflicting injury to the genital tract. He always emptied the uterine cavity at the earliest possible period.

Dr. T. D. Fitch did not consider Dr. Sawyer's specimen a rare pathological occurrence. He had seen the same separate,—at the same time,—frequently. He agreed with Dr. Nelson in treatment. Still, when the cervix was not dilated, he was disposed to prefer the expectant line of treatment. At the time of the occurrence of abortion, the uterus was in a physiological condition; operative interference at a later period was attended by increased risk, as the utérus was then in a pathological state. Usually, he found the placenta and membranes detached within the lower segment of the uterus. As he had a large hand, with short fingers, he employed the placental forceps of Hodge, Dewees, Elliott or Roler. Once, in the country, he used an ordinary pail bail, with the rough edges filed off. He was not in favor of intra-uterine injections. The vaginal douche was sufficient.

Dr. Wm. H. Byford said that abortions were more frequent in large cities than in the country. Madame La Chapelle's estimate of the frequency of abortion was not exaggerrated, when applied to large communities. In the country, he thought that one abortion to three labors at term represented a fair average. Abortion was never a physiological process, although abortions from disease of the ovum were attended with less danger than those resulting from morbid, uterine changes. In disease of the ovum, the circulation was impaired, the embryo perished, and expulsion follows, with the minimal degree of hemorrhage, pain and sepsis. When the cause of abortion was external violence, or decidual endometritis, dangēr, in each of these three directions, was increased. The specimen exhibited by Dr. Sawyer, was of rare occurrence at so late a period; it was comparatively common during the early months.

As regards the prophylactic treatment, he had observed two clinical facts, in connection with the habit of abortion. When uterine contractions were the prominent symptom, abortion could be arrested in many cases by absolute rest and opium; when hemorrhage was severe, all attempts at arresting the process were usually futile. This was especially true during the first three months. At a later period, even when hemorrhage was severe, abortion might be arrested.

In regard to the treatment of inevitable abortion, he had never seen the time, when champions of the expectant and radical courses of action did not exist. The treatment must be governed by the consideration of the individual case. In any case, the patient must be carefully watched. He had never seen a case of abortion terminating by immediately fatal hemorrhage. The acute anæmia, however, might induce a condition which would render the woman more susceptible to sepsis or any intercurrent disease. He feared sepsis and metro-peritonitis more than hemorrhage. He was conservative as to the operative interference. Let nature do what she can; only in case of failure on her part, interfere. The finger was preferable to any instrument. Then it was not necessary to insist upon the removal of placenta or membranes with mathematical accuracy. If the placenta was grasped by an irregularly contracted uterus, cut off the free portion, allow the rest to remain in the uterine cavity. If two-thirds of the placenta were removed and the uterus well contracted, the case was to be considered in a safe condition. In event of sepsis, remove all the intrauterine mass.

Dr. John Bartlett stated that, in his practice abortions were as frequent as labors at term. The product of conception was usually expelled in its integrity. When abortion was inevitable two conditions were required, before operation interference was justifiable.

(1.) Dilatation of the canal of the cervix to the extent necessary for the passage of two fingers. (2.) The separation between decidua and the uterine mucosa must be more or less complete. Until these conditions were present, the vagina ought to be tamponed. For a tampon, he was in the habit of employing bits of cotton tied on a string, in the manner of the kite-tail.

He had seen cases in which the placenta had remained for a long period of term within the uterine cavity without causing symptoms. The retention in one case lasted through a period of three months; in the other case, the period was four months. As to the length of time, the tampon could be left *in situ*, he had adopted Dr. De Laskie Miller's rule of allowing it to remain twenty-four hours. He had frequently tamponed through three days. The colpeurynter of Braun was a useful tampon. No tampon was effective when the hemorrhage was not of a passive character, and uterine contractions were severe.

It was necessary to diagnosticate between placenta prævia and abortion. Placenta prævia implied simply an *error loci* of the ovum. It was included between the ring of Bandl and the external os. Dr. Bartlett had never seen a case of abortion terminate fatally from hemorrhage.

Dr. A. H. Foster had seen one case of retained placenta, in which the retained mass gave origin to no serious symptoms for a period of four months. The importance of subsequent treatment of the puerperium was urged.

Dr. E. C. Dudley referred to the dangers of cervical laceration, and subinvolution in consequence of abortion. The best method of applying the tampon was by means of Sim's speculum.

Dr. C, W. Earle occupied the middle ground between the expectant and the radical methods of treatment. He did not agree with Dr. Mundé.

Dr. H. P. Merriman; after endorsing Dr. Byford's remarks on the ætiology of the condition, said that he did not like to use ergot in these cases, as it caused irregular contraction of the internal os, imprisoning the placenta without favoring its expulsion. He produced uterine contractions by dilating the os, and then followed the expectant plan of treatment.

Dr. E. W. Sawyer, in closing the discussion, said that he had observed abortion in the lower animals, and concluded nature required little interference. He then briefly sketched the line of expectant treatment, which he was in the habit of recommending.

Dr. John Bartlett then exhibited some casts of the pregnant and non-pregnant uterus for the purpose of class illustration. The idea is an extremely ingenious one, and will receive attention at an early period.

The Society adjourned, to meet Dec. 19th, at the residence of the President, Dr. H. P. Merriman, No. 1350 Michigan avenue. Subject for discussion, "Extra-Uterine Pregnancy," to be introduced by Dr. Wm. H. Byford by a paper on "A Case of Interstitial Pregnancy."

W. W. JAGGARD, M. D., *Editor*,

November 22nd, 1884. 2330 Indiana ave.

